# Association of New-Onset Atrial Fibrillation With All-Cause Mortality in COVID-19 Patients

**DOI:** 10.7759/cureus.49785

**Published:** 2023-12-01

**Authors:** Kamran Zaheer, Bruno Goncalves, Archana Ramalingam, Noor Ul Ann Rabbani, Rameez Sayyed, Athar Nawab, Raghav Puri, Charles J Williams, Kanaan Mansoor

**Affiliations:** 1 Department of Internal Medicine, St. Mary's Medical Center, Huntington, USA; 2 Department of Surgery, Marshall University Joan C. Edwards School of Medicine, Huntington, USA; 3 Department of Internal Medicine, Marshall University Joan C. Edwards School of Medicine, Huntington, USA; 4 Department of Cardiology, Marshall University Joan C. Edwards School of Medicine, Huntington, USA

**Keywords:** outcome, atrial fibrillation, arrhythmia, cardiovascular complication, covid-19

## Abstract

Background

The COVID-19 pandemic has brought about unprecedented global health challenges, with its impact extending beyond respiratory manifestations to encompass cardiovascular complications, including arrhythmias. Dysrhythmias in COVID-19 are multifactorial, ranging from direct myocardial insult due to the cytokine storm to metabolic derangements.

Objective

In this study, we aim to examine the incidence of new-onset atrial fibrillation and to study its association with all-cause mortality of COVID-19.

Methods

A cross-sectional study was conducted at Cabell Huntington Hospital, West Virginia, utilizing electronic medical records of COVID-19 patients from 2020 to 2021. Inclusion criteria comprised patients aged >18 years with COVID-19 diagnosis and cardiac arrhythmias during hospitalization. Logistic regression analysis was employed to examine the relationship between demographic and clinical variables and in-hospital mortality.

Results

Of the 264 eligible patients, those aged >66 years had lower odds of in-hospital mortality (p < 0.001), while gender, ejection fraction, and diabetes mellitus did not significantly predict mortality. Atrial fibrillation (p = 0.011) and heart failure (p = 0.030) were associated with increased odds of mortality, while hypertension showed no significant predictive power (p = 0.791).

Conclusion

This study highlights the significance of atrial fibrillation and heart failure as predictors of in-hospital mortality in COVID-19 patients. Our findings underscore the importance of recognizing and managing arrhythmias in COVID-19 and call for further research on the mechanisms and long-term effects of these cardiac complications in the context of the pandemic. These insights can guide clinical practice and interventions to optimize patient outcomes.

## Introduction

Coronavirus disease 2019 (COVID-19) has rapidly transformed into a global health crisis of unprecedented proportions, affecting millions of individuals worldwide [1,2]. In 2020, the COVID-19 pandemic resulted in over 490,000 cases and 18,500 deaths in the United States, with all 50 states affected [1]. Initially characterized as a contagion primarily affecting the respiratory system, COVID-19 subsequently unveiled its intricate and multisystemic pathophysiology, thereby extending its deleterious consequences far beyond the confines of pulmonary affliction [3]. The pathogenesis of SARS-CoV-2 infection involves the molecular interaction between the viral surface S-protein and the angiotensin-converting enzyme 2 (ACE2), serving as the critical receptor molecule. ACE2 predominantly resides within the pulmonary milieu, primarily serving as the principal portal of entry for the virus. It is notable that ACE2 exhibits a substantial presence within the cardiac domain, thereby predisposing individuals to the potential development of cardiovascular complications [4,5].

It is known that COVID-19 may cause cardiac involvement at macrovascular and microvascular levels [6]. Numerous cardiovascular complications have been identified in the context of COVID‐19, including myocarditis, ST-elevation myocardial infarction (STEMI), arrhythmias, vascular endothelial dysfunction, thromboembolism, coronary vasospasm, and cardiomyopathy, all of which exhibit a strong association with adverse clinical outcomes [6-8]. The incidence of these complications exhibits substantial heterogeneity across different study populations, with arrhythmia emerging as the second most prevalent complication following acute respiratory distress syndrome (ARDS) [9]. Arrhythmia is characterized by abnormalities or perturbations in the normal activation or beating of the heart myocardium [10]. The documentation of arrhythmias in patients with SARS‐CoV‐2 infection has been somewhat limited; however, a cumulative incidence of 16.7% for arrhythmia development has been reported among hospitalized COVID-19 patients [11]. Furthermore, arrhythmias were observed in 7% of non-ICU-treated patients and in a staggering 44% of individuals requiring ICU admission [12]. The most common arrhythmia overall in patients with COVID-19 is sinus tachycardia; however, the most frequent pathological arrhythmias include atrial fibrillation, atrial flutter, and monomorphic or polymorphic ventricular tachycardia [9,13]. These conditions are relevant manifestations that lead to a significant reduction in the quality of life of the patients.

The unexpectedly high prevalence of cardiac arrhythmias after COVID-19 requires careful exploration of the pathology leading to myocardial injury and, subsequently, an increase in myocardial electric instability. Overall, the mechanisms related to the development of arrhythmias in SARS-CoV-2 infected patients are not fully elucidated; however, the potential pathophysiological mechanisms underlying arrhythmias encompass (1) direct injury to cardiomyocytes disrupting both the integrity of the plasma membrane and the normal electrical conduction pathways [14]; (2) potential infection of the pericardium, instigating significant edema [15]; (3) ischemia from microvascular disease due to possible infection of the pericytes [16,17]; (4) the induction of re-entrant arrhythmias attributable to myocardial fibrosis [18]; (5) the influence of proinflammatory cytokines, rendering a predisposition to arrhythmogenicity [19]. Scenarios 1, 2, and 3 may manifest acutely, while scenarios 4 and 5 typically occur in a chronic or post-inflammatory myocarditis context. In scenario 5, proinflammatory cytokines such as IL-6 could potentially induce the displacement of plakoglobin, a desmosomal protein, from the cardiomyocyte membrane [20]. This has the potential to be arrhythmogenic, as it may result in insufficient cell-to-cell adhesion, postulated to cause damage to the cell membrane, subsequently leading to cardiac cell demise and fibrofatty tissue replacement [21]. Furthermore, the reduced surface expression of desmosomal proteins represents a recognized etiological factor in arrhythmogenic cardiomyopathies [21]. Therefore, it is plausible that SARS-CoV-2 infection precipitates arrhythmias in patients with a genetic predisposition.

The COVID-19 pandemic has been marked not only by its clinical complexity but also by its profound impact on global health and society. This study, therefore, aims to examine the incidence of new-onset atrial fibrillation and to study its association with all-cause mortality of COVID-19 in West Virginia. The findings from this study can provide valuable insights into the clinical implications of arrhythmias in COVID-19 patients and potentially guide healthcare providers in implementing appropriate interventions to optimize patient outcomes.

## Materials and methods

Study design

This cross-sectional study was performed at Cabell Huntington Hospital, Huntington, West Virginia (WV). This hospital is a large rural medical center with a preponderance of COVID-19 patients from WV between 2020 and 2021. The study was completed by retrospective chart review of patient’s electronic medical records (EMR). The study was approved by the institutional review board (IRB) of Marshall University Joan C. Edwards School of Medicine, Huntington, WV (IRB No: 1735588), and a waiver was obtained for informed consent.

Patient selection and data collection

To ensure an appropriate selection of patients eligible for the study, trained hospital personnel examined patient’s EMRs with appropriate confidentiality measures and in compliance with the Health Insurance Portability and Accountability Act of 1996 (HIPAA). Specifically, patients were identified by the review of EMR using the search period from 2020 and 2021. During this time, patients aged >18 years old, admitted to the hospital with a clinical diagnosis of COVID-19, and who developed any cardiac arrhythmias during their hospital stay were identified and included in the study. For the exclusion criteria, all patients under 18 years old, pregnant, and patients with COVID-19 who did not develop any cardiac arrhythmia during their hospital stay were excluded from this study. Patients meeting the inclusion criteria were included and relevant variables, such as age, gender, ejection fraction (EF), presence of atrial fibrillation (AF), presence of comorbid conditions, including diabetes mellitus and hypertension, and outcome (mortality), were collected.

Statistical analysis

Data were analyzed using IBM SPSS Statistics version 26 software (IBM Corp., Armonk, NY). Demographic and clinical variables are presented as frequency/percentage. Categorical variables were employed in logistic regression analysis to explore the association between independent variables and the dependent variable, i.e., “death in the hospital.” All data comparisons are presented as p < 0.05 (confidence interval of 95%) and p < 0.01 (confidence interval of 99%).

## Results

Demographics

A total of 264 patients met all the inclusion criteria for the diagnosis of COVID-19 and the development of any kind of cardiac arrhythmia during their stay at Cabell Huntington Hospital, Huntington, WV. These patients were selected from a specific rural population, which provided unique insights into this demographic. Table 1 summarizes the basic characteristics of the study population as well as comorbidities in the population within each group. The quantitative values are shown as a percentage of each group's population. For the age variable, participants were categorized into two age groups. The majority, 160 individuals (60.5%), were aged > 66 years. The gender distribution in the study was relatively balanced, with 154 males (57.9%). In terms of hypertension (HTN), a notable proportion of participants, 76 individuals (28.6%), reported having HTN. EF% values were divided into two categories. The majority, 234 individuals (88.3%), had EF% values between 50 and 70. AF status was assessed, with 118 individuals (44.7%) presenting with AF. Among the participants, 135 individuals (51.1%) had diabetes mellitus (DM). Regarding heart failure (HF), a significant proportion of participants, 111 individuals (42.1%), had a diagnosis of HF. This comprehensive demographic dataset is crucial for understanding the characteristics of the study population and will be used to assess the impact of these demographic factors on the incidence of arrhythmias and overall mortality rates among COVID-19-diagnosed individuals in West Virginia.

**Table 1:** Summary of patient demographics and general clinical profile.

Category	Frequencies (N = 264)	Percentage (%)
Age (>66)	160	60.5
Gender (male)	154	57.9
Hypertension	76	28.6
Ejection fraction (EF%): 50-70	234	88.3
Atrial fibrillation	118	44.7
Diabetes mellitus	135	51.1
Heart failure	111	42.1

Logistic regression analysis

Table 2 reports the results of a logistic regression analysis that investigates the relationship between multiple independent binary outcome variables and the dependent variable, i.e., in-hospital mortality (outcome). The independent variables under examination consist of age group, gender, EF group, AF group, DM, HF, and HTN. Age variable demonstrates a highly significant association with in-hospital mortality (p < 0.001). The odds ratio (Exp (B)) is 0.282, with a 95% confidence interval (CI) ranging from 0.149 to 0.532. This implies that for each unit increase in the age group, the odds of in-hospital mortality decrease by a factor of 0.282, indicating that younger age is associated with lower odds of mortality. Gender (male and female) does not exhibit a statistically significant association with in-hospital mortality (p = 0.232). The odds ratio is 1.404, and the 95% CI spans from 0.805 to 2.449. Therefore, gender does not appear to be a strong predictor of in-hospital mortality. In terms of the EF group, there is no statistical significance in predicting in-hospital mortality (p = 0.73). The odds ratio is 0.458, and the 95% CI ranges from 0.196 to 1.074. Consequently, the EF group does not have a substantial impact on the odds of in-hospital mortality. AF group demonstrates a statistically significant association with in-hospital mortality (p = 0.011). The odds ratio is 2.163, with a 95% CI spanning from 1.193 to 3.920. This indicates that individuals in the AF group have significantly higher odds of in-hospital mortality compared to those not in the AF group. DM does not show a statistically significant association with in-hospital mortality (p = 0.528). The odds ratio is 0.836, and the 95% CI ranges from 0.479 to 1.459. Therefore, the presence or absence of DM does not strongly predict in-hospital mortality. HF presents a statistically significant association with in-hospital mortality (p = 0.030). The odds ratio is 1.925, and the 95% CI ranges from 1.064 to 3.484. This suggests that individuals with HF have elevated odds of in-hospital mortality. HTN does not exhibit a statistically significant association with in-hospital mortality (p = 0.791). The odds ratio is 0.921, and the 95% CI spans from 0.502 to 1.690. Thus, HTN is not a strong predictor of in-hospital mortality.

**Table 2:** Logistic regression analysis of independent binary outcome variables predicting in-hospital mortality.

Variables	Significance (p-value)	EXP (B)	95% CI for EXP (B)	
			Lower	Upper
Age	<0.001	0.282	0.149	0.532
Gender	0.232	1.404	0.805	2.449
Ejection fraction	0.073	0.458	0.196	1.074
Atrial fibrillation	0.011	2.163	1.193	3.920
Diabetes mellitus	0.528	0.836	0.479	1.459
Heart failure	0.030	1.925	1.064	3.484
Hypertension	0.791	0.921	0.502	1.690
Constant	0.37	2.442		

## Discussion

Since the beginning of the pandemic, it has rapidly become evident that acute infection is not limited to the respiratory tract but that several organs, including the cardiovascular system, emerging as an ongoing challenge for physicians and healthcare professionals [8]. Several cardiovascular complications in COVID-19 have been identified, with arrhythmia recognized as the second most common complication after acute respiratory COVID-19 infection and is involved in fatal outcomes [6,9]. Therefore, identifying the mechanism underlying arrhythmias in COVID-19 and their implications for patient outcomes is crucial for optimizing clinical management strategies.

Our observation of AF as a significant predictor of in-hospital mortality aligns with a growing number of studies highlighting the association between COVID-19 and cardiac arrhythmias [22-24]. Recent studies have demonstrated that SARS-CoV-2 infection can lead to direct myocardial injury, inflammation, and autonomic dysfunction, all of which can promote the development of AF [25-27]. Furthermore, emerging research has elucidated the molecular mechanisms underlying this relationship, including the role of viral entry receptors (e.g., ACE2) in cardiac tissue and the subsequent proinflammatory cytokine cascade [28-31]. In addition, studies have reported a higher incidence of atrial arrhythmias in COVID-19 patients and identified AF as an independent predictor of mortality [32,33]. Similarly, in a cross-sectional study, the incidence, mortality, and imaging outcomes of atrial arrhythmias in COVID-19 patients reported that atrial arrhythmias were associated with markers of inflammation and cardiac injury [2]. These findings underscore the multifaceted nature of COVID-19's impact on the cardiovascular system, warranting continued investigation into therapeutic strategies targeting arrhythmia management in COVID-19 patients.

Previously, older age has been reported as an important independent predictor of mortality in severe acute respiratory syndrome (SARS) [34]. The current study revealed a significant and protective effect of younger age on in-hospital mortality among COVID-19 patients. This finding concurs with extensive literature highlighting the heightened vulnerability of older individuals to severe COVID-19 outcomes [35,36]. It has linked age-related changes in the immune system, such as immunosenescence and inflammation to increased susceptibility to severe disease [35]. Additionally, the age-dependent defects in T-cell and B-cell function and the excess production of type 2 cytokines could lead to a deficiency in control of viral replication and more prolonged proinflammatory responses, potentially leading to poor outcomes [37]. The inflammation led by the virus contributes to vascular inflammation, myocarditis, and cardiac arrhythmias as well [38,39].

It is important to note that arrhythmias in COVID-19 patients may also be influenced by the medications used in the management of the disease [40]. Certain medications, such as those used for early COVID-19 treatment, may contribute to arrhythmias, primarily QT prolongation [41]. Monitoring drug-drug interactions and being cautious with the use of medications in COVID-19 patients is crucial to prevent potential arrhythmogenic effects and other effects that could lead to future HF. Our study identified HF as another significant predictor of in-hospital mortality among individuals with COVID-19. This result is consistent with previous reports highlighting the vulnerability of heart failure patients to adverse outcomes in the setting of viral infections [42-44]. COVID-19-induced systemic inflammation and myocardial injury can exacerbate HF and lead to decompensation [31,45,46]. The intricate molecular mechanisms underlying HF remain incompletely understood. Studies have explored factors contributing to HF, encompassing direct injury to cardiomyocytes that disrupt both the integrity of the plasma membrane and the normal electrical conduction pathway [11]. Additionally, potential pericardial infection has been implicated, leading to significant edema [12]. Microvascular disease-induced ischemia, possibly stemming from pericyte infection, is considered [12-14]. The induction of re-entrant arrhythmias, attributed to myocardial fibrosis, is another identified factor [18]. Dysregulation of immune responses, cytokine storms, and oxidative stress further contribute to HF pathophysiology. These factors possess arrhythmogenic potential by potentially compromising cell-to-cell adhesion, hypothesized to induce damage to the cell membrane and subsequent cardiac cell demise, leading to fibrofatty tissue replacement [21,29-31,47]. Understanding these molecular mechanisms is essential for developing targeted interventions to mitigate the impact of HF on COVID-19 mortality. Additionally, our findings underscore the importance of close monitoring and tailored management for COVID-19 patients with pre-existing heart failure.

Contrary to some reports, our data did not find gender, EF group, DM, or HTN to exhibit strong predictive power for in-hospital mortality among COVID-19 patients [42,43,48,49]. These findings align with the growing number of studies indicating that the relationships between these variables and COVID-19 outcomes are complex and multifactorial. In addition, it is important to consider the specific population studied. While gender-based differences in COVID-19 outcomes have been observed in various studies, the exact mechanisms underlying these disparities remain debated and may be influenced by a myriad of factors [50-52]. Similarly, the predictive value of EF alone for COVID-19 mortality may be attenuated by the multifaceted cardiac effects of the virus, including viral-induced inflammation and myocardial injury. Moreover, the absence of robust associations between DM and HTN with COVID-19 mortality in our study highlights the intricate interplay of comorbidities and the need for considering molecular and systemic factors in understanding these relationships.

**Figure 1:**
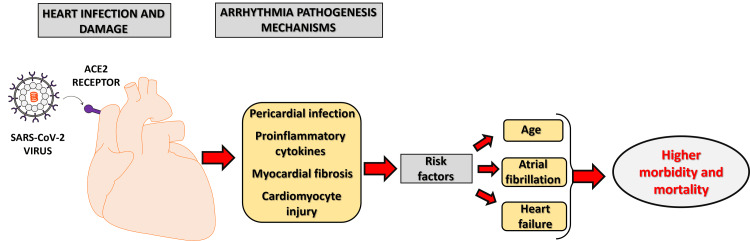
Schematic representation showing the complications of COVID-19 leading to arrhythmia and factors involved in in-hospital mortality. ACE2: angiotensin-converting enzyme 2. Image proposed by the author.

However, the study has limitations. The main limitation of this study is the relatively small sample size. In addition, research is warranted to validate these results, explore additional factors influencing mortality in COVID-19 patients with atrial fibrillation, and account for potential variables such as obesity and laboratory values that were not included in our study. Despite these limitations, this study presents important strengths. To our knowledge, no previous study has investigated the incidence of arrhythmias among individuals diagnosed with COVID-19 in West Virginia and their impact on overall mortality rates. Therefore, we believe that the unique nature of the rural population studied is a strength of this analysis, providing insights into a specific and often understudied demographic. Figure 1 summarizes the complications associated with COVID-19 and arrhythmia, as well as the factors that influence in-hospital mortality.

## Conclusions

In conclusion, our study has contributed valuable insights into the factors influencing in-hospital mortality among COVID-19 patients in West Virginia. The significance of AF and HF as predictors highlights the critical role of cardiac rhythm disturbances in COVID-19. In addition, these data will inform clinical practice and guide future research endeavors in the quest to mitigate the impact of this global pandemic on vulnerable patient populations. Further investigations are warranted to elucidate the precise mechanisms underlying the relationship between arrhythmias and COVID-19 mortality and exploring the potential cardiac sequelae beyond the acute phase of the disease will be crucial for optimizing therapeutic strategies aimed at reducing mortality rates in this context.
